# Nurturing care assets for food security: a community asset mapping approach

**DOI:** 10.1186/s41043-024-00573-9

**Published:** 2024-06-06

**Authors:** Gabriela Buccini, Kaelia Lynn Saniatan, Ana Poblacion, Ana Bauman, Cristina Hernandez, Cali Larrison, Dodds P. Simangan, Jyoti Desai, Yvonne Owens Ferguson, Alisa Howard, Melva Thompson-Robinson

**Affiliations:** 1 Department of Social and Behavioral Health, School of Public Health, University of Nevada Las Vegas, Las Vegas, NV USA; 2 Department of Pediatrics, Chobanian & Avedisian School of Medicine, Boston University, Boston, MA USA; 3 Department of Pediatrics, Boston Medical Center, Boston, MA USA; 4 Division of Public Health Sciences, Department of Surgery, Washington University School of Medicine, St. Louis, MI USA; 5 Department of Pediatrics, Kirk Kerkorian School of Medicine, University of Nevada Las Vegas, Las Vegas, NV USA; 6 Department of Gynecologic Surgery and Obstetrics, Kirk Kerkorian School of Medicine, University of Nevada Las Vegas, Las Vegas, NV USA; 7 Office of Strategic Coordination, Division of Program Coordination, Planning, and Strategic Initiatives, Office of the Director, National Institutes of Health, Bethesda, MD USA; 8 Minority Health Consultants, Las Vegas, NV USA; 9 School of Nursing, University of Nevada Las Vegas, Las Vegas, NV USA

**Keywords:** Community asset mapping, Food security, Maternal-child health, Nutrition

## Abstract

**Background:**

Zip codes classified by the Food Insecurity Index with moderate and high food insecurity (FI) risk can be a threat to the health and well-being of children during the first 1,000 days (from pregnancy to 2 years). The presence of nurturing care assets (i.e., stable environments that promote health and nutrition, learning opportunities, security and safety, and responsive relationships) can contribute to supporting families and their communities, and ultimately reduce systemic barriers to food security. We aimed to identify and characterize nurturing care assets in under-resourced communities with moderate and high FI risk.

**Methods:**

Four steps were used to conduct a Community Asset Mapping (CAM): (1) review of community documents across five zip codes in Clark County, Nevada (2), engagement of community members in identifying community assets (3), definition of the assets providing nurturing care services, and (4) classification of assets to nurturing care components, i.e., good health, adequate nutrition, safety and security, opportunities for early learning, and responsive caregiving. The Food Insecurity Index was used to determine FI risk in each zip code. Analyses explored whether disparities in nurturing care assets across zip codes with moderate and high FI exist.

**Results:**

We identified 353 nurturing care assets across zip codes. A more significant number of nurturing care assets were present in zip codes with high FI risk. The adequate nutrition component had the most assets overall (*n* = 218, 61.8%), while the responsive caregiving category had the least (*n* = 26, 7.4%). Most of the adequate nutrition resources consisted of convenience stores (*n* = 96), food pantries (*n* = 33), and grocery stores (*n* = 33). Disparities in the number and type of good health, early learning, and security and safety assets were identified within zip codes with high FI risk compared to moderate FI risk.

**Conclusions:**

The quantity and type of nurturing care assets can exacerbate existing demographic disparities across zip codes, which are tied to barriers to access to food in under-resourced communities in Clark County, Nevada. Co-creating a nurturing care asset-based zip code strategy to address high FI risk will require strengthening systems across existing nurturing care assets.

**Supplementary Information:**

The online version contains supplementary material available at 10.1186/s41043-024-00573-9.

## Background

Many of the social determinants of health can be traced back to the zip code in which one lives [1–3]. Food insecurity (FI) is a determinant of health described as a lack of consistent access to enough healthful food for an active, healthy life due to financial constraints [2, 4, 5]. The Food Insecurity Index (FII) is a validated measure of economic and household hardship correlated with poor food access for all zip codes across the U.S [5]. Zip codes with moderate and high FII values are estimated to have high socioeconomic needs correlated with FI risk, which in turn negatively influence health, especially during the first 1,000 days from pregnancy to two years of a child’s age [6].

Systemic barriers in moderate and high FI risk zip codes may prevent pregnant people and parents of young children from providing the nurturing care that they need to thrive (i.e., stable and safe environments that promote health and nutrition, learning opportunities, and responsive relationships) [7, 8]. Specifically, FI is associated with multiple overlapping unmet social needs such as housing instability, high utility costs, medical costs, lack of transportation, unemployment, poverty, and lifetime racial discrimination that threatens optimal development during the first 1,000 days (from pregnancy to age 2 years) [9, 10]. FI increases the risk of adverse outcomes during pregnancy such as maternal depression and anxiety, malnutrition, and anemia [11], as well as infant health and development, such as low birth weight [12, 13], chronic undernutrition and obesity [14], increased infectious diseases, and impaired cognitive development [15]. As a result, these disproportionate negative impacts on child development in under-resourced zip code areas may perpetuate the cycle of poverty [3, 16].

Effective interventions to address FI across under-resourced zip code areas need to consider the interplay between multilevel socio-ecological factors, such as political, economic, and social drivers of inequities that shape zip code areas with a high prevalence of FI. While no unique solution exists to address FI, community-level strategies that prioritize the zip codes where under-resourced families reside can play a significant role in reducing systemic barriers and ultimately inequities [17]. Accordingly, the Nurturing Care Framework (NCF) provides a roadmap of five components (i.e., good health, adequate nutrition, early learning, security and safety, and responsive caregiving, [see Table 1 for operational definitions]) to transform child rights into equitable actions to promote optimal development during the first 1,000 days [18–20]. We hypothesized that the presence of nurturing care assets such as reliable childcare facilities, food retailers, safe after-school activities, and healthy recreational amenities may be assets to address the structural drivers of FI [15, 19, 21, 22]. In this study, we utilize the NCF as a theory to conceptualize a multi-sectoral approach to address FI in under-resourced zip code areas. To our knowledge, a nurturing care asset-based strategy to promote equity and food security during the first 1,000 days has not been described.

**Table 1:** Types of assets considered within nurturing care components

Nurturing Care Component	Tailored definitions of the nurturing care components to the context of zip code	Types of Assets
**Good Health**	Refers to the existence of assets, in a given zip code, to promote the overall health, mental health, and well-being of children and caregivers.	• Hospitals • Urgent Cares • Mental Health Centers • Outreach Clinics • Pediatric Health Clinics • Women’s Health Care Centers • Non-Profit Health Organizations
**Adequate Nutrition**	Refers to the existence of assets, in a given zip code, to promote access to healthy food and nutrition education.	• Food Pantries • Food Distribution Sites • Grocery Stores • Convenience Stores • Farmers Markets • Maternal and Child Food Programs (WIC) • Community Gardens
**Security and Safety**	Refers to the existence of assets, in a given zip code, to promote safe and secure environments (i.e., physical dangers, emotional stress, environmental risks, and access to food and water).	• Police Stations • Public Parks • Libraries • Churches • Community Centers
**Opportunities for Early Learning**	Refers to the existence of assets, in a given zip code, to promote access to childcare and early education laying the foundation for later learning.	• Preschools • Daycares • Non-Profit Education Programs • Education Financial Subsidies
**Responsive Caregiving**	Refers to the existence of assets, in a given zip code, to support caregivers’ ability to respond to the signals of children in a timely and appropriate manner.	• Doulas • Lactation Specialists • Home Visiting Programs

Community asset mapping (CAM) is an approach where community members are engaged to identify and document available assets that are likely to address intervention goals. Assets are institutions, individuals, and citizen associations that serve as a support system [22, 23]. CAM was created to change the perspectives of people to see zip code areas with assets as strengths rather than deficits [24, 25]. This method provides valid and reliable data on the strengths of a zip code area and on the community’s ability to problem solve [26]. Our study aimed to use CAM to identify and characterize nurturing care assets that provide care to pregnant people, caregivers, and their infants in under-resourced zip code areas with moderate and high FI risk.

## Methods

### Study design

This exploratory study utilized CAM through multi-collaborative efforts among the research team, community-partnered organization, and community members to identify and characterize nurturing care assets in under-resourced zip code areas with moderate and high FI risk in Clark County, Nevada, U.S.

### Study setting

This study takes place in five zip codes located in Clark County, Nevada, U.S. These zip codes were selected because they are part of the “West Las Vegas Promise Neighborhood (WLVPN).” WLVPN is a place-based social initiative implemented in 2011 by a non-profit organization named Nevada Partners, that coordinated with over 50 multi-sector partners in five zip codes across central Clark County, Nevada to reduce structural inequities [27]. These five zip codes include 89,101, 89,106, 89,030, 89,031, and 89,032, corresponding to under-resourced and historically African American zip codes in Clark County [28]. Approximately 260,000 residents inhabit these zip codes with an average of 14.3% of families with children living below the poverty line [29]. In addition, most of the residents identify as Hispanic/Latinx (~ 50.0%), some other race (26.6%), and Black/African American (23.6%) [28] (Appendix 1).

### Systematic community asset mapping process

Four steps were taken to complete the community asset mapping process.

#### Review of community documents

The research team searched for community assets providing maternal-child health and nutrition related services within the identified zip codes. First, we reviewed documents of organizations providing health services provided by our community partner. Second, we conducted a web search of established businesses and programs within the identified areas. Along with web-engine searches included contacting these assets to confirm their business was active. Third, as an additional effort to identify assets, the research team included organizations or professionals that travel to provide services within the identified zip codes. We specifically looked for midwives (i.e., Certified Nurse Midwives (CNM), Certified Professional Midwives (CPM), and Traditional Midwives), doulas, lactation consultants, childbirth educators, and support groups for the prenatal and postpartum periods. Most individuals in these professions do not have their own office space. We developed a survey with 13 questions to identify the location of provided services. Questions included whether the doula/birth worker was currently practicing in the zip codes, and if so their schedule (full-time/part-time), focus population (e.g., LGBTQIA + and BIPOC), and number of clients they have served within the identified zip codes. The survey was disseminated to the large community of doula/birth workers via social media and intended contacts (Appendix 2). All identified assets were compiled into an Excel database (NCF assets database, version 1).

#### Engagement of community members in identifying community assets

**Fig. 1:**
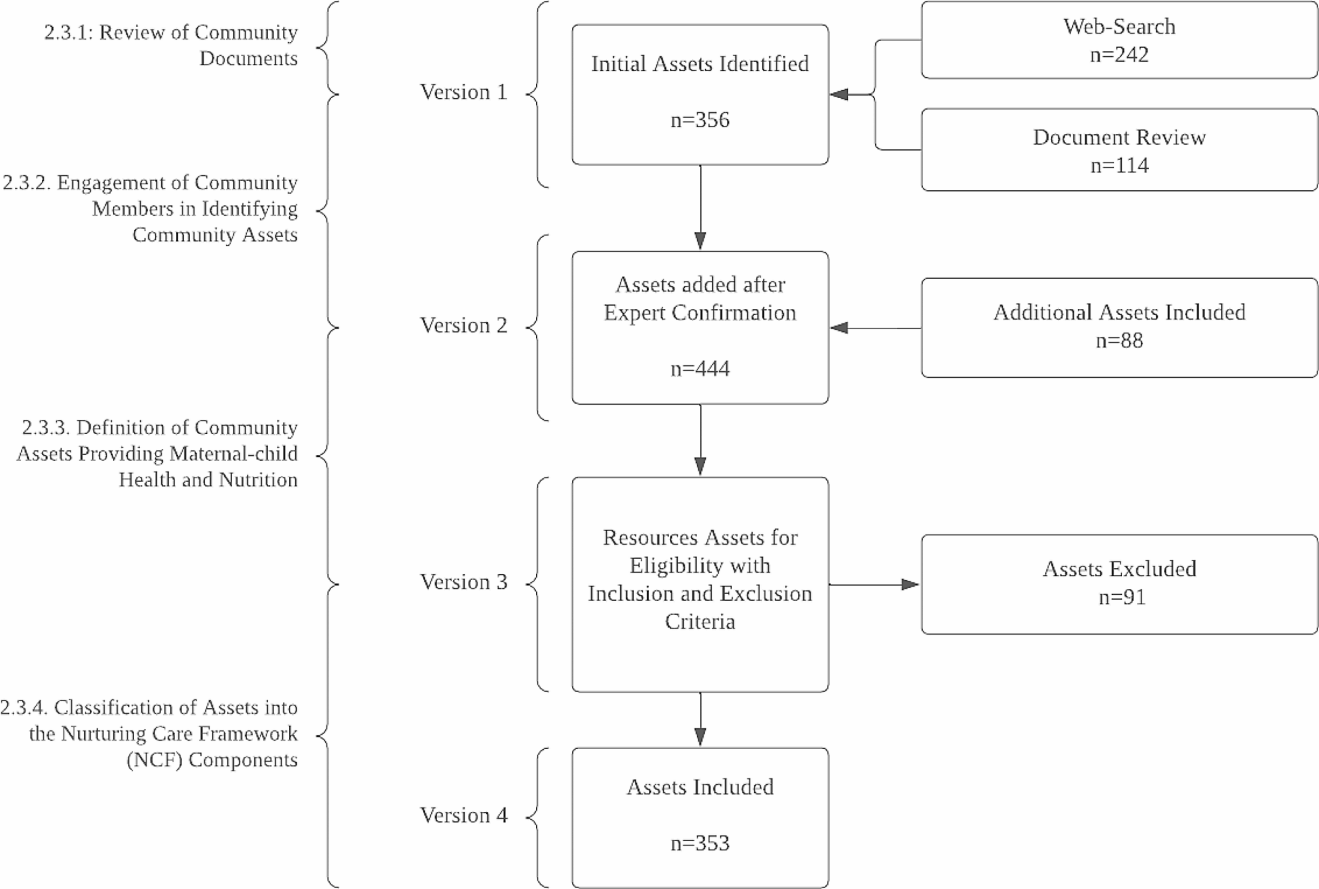
Flow chart of the systematic mapping of assets process

We engaged with two long-standing members of the community to interactively identify the assets compiled into the Excel database (version 1). The first member is a minority health consultant, licensed community health worker, and health educator. The second member is a researcher in health disparities and equity who had a long relationship working within the African American community in the “West Las Vegas Promise Neighborhood”. Both community members received instructions about the objective of the CAM and procedures to confirm, remove, or include assets. Using the Excel database (version 1), both members met and through discussions confirmed the assets. In addition, they reached out to their existing networks within the community to search for any missing assets. One of the members visited organizations to determine if they provided services to the population of focus for the project and attended community meetings to learn about the organizations. Along with visiting the organizations, the members also conducted a windshield survey [30], i.e., drove by the identified zip codes to include assets that could not be located or reached by phone or website. Any new assets identified were placed into the second version of the Excel database (version 2). Variations between Excel database versions 1 and 2 are displayed in Fig. 1.

#### Definition of community assets providing nurturing care services

To be included in the nurturing care asset mapping, a community asset must provide nurturing care services within the identified zip codes or travel to pregnant people, infants, and toddlers living within these zip codes. Eligibility criteria were developed based on the NCF definitions for each component (Table 1). Nurturing care services may include, but are not limited to, childcare facilities, food retailers and distribution sites, community centers, public libraries, and more. For an asset to be excluded from the mapping, it must not focus on nurturing care, such as a lack of focus on pregnancy and early childhood. After applying the eligibility criteria, a third version of the Excel database (version 3) was generated.

#### Classification of community assets into the nurturing care framework (NCF) components

We operationalized the definitions of the NCF components as follows (1) Good Health (assets to promote the overall health, mental health, and well-being of children and caregivers) (2), Adequate Nutrition (assets to promote access to healthy food and nutrition education) (3), Security and Safety (assets to promote safe and secure environments) (4), Opportunities for Early Learning (assets to promote access to childcare and early education laying the foundation for later learning), and (5) Responsive Caregiving (assets to support caregivers’ ability to respond to the signals of children in a timely and appropriate manner) (see Table 1 for detailed description). Based on these operational definitions, two members of the research team (KS, CL) using the third version of the Excel database (version 3) classified the assets into one of the five components of the NCF. A senior research expert in NCF application (GB) reviewed the classification, and any discrepancies were discussed until a consensus was reached (version 4).

### Measurements

#### Nurturing care assets

Nurturing care assets identified through the systematic community asset mapping process in the final Excel database (version 4) were the independent measure of data analysis. We hypothesized that the higher the risk for FI the higher the need for community assets across each NCF component in under-resourced zip codes.

#### Food Insecurity Index (FII)

The FII is the dependent measure of data analysis, and it was selected for its ability to measure FI risk considering the social and demographic characteristics on a zip code level. FII is a validated tool developed by the Conduent Healthy Communities Institute to measure of economic and household hardship correlated with poor food access. The FII is estimated by combining indicators across four topics: Financial Stability, Household Environment, Medicaid enrollment, and Wellness into a single composite value [5]. As a single indicator, the index can serve as a concise way to identify which areas are of the highest need. Detailed information on how the index was created and validated is reported elsewhere [5]. Based on FII calculation, zip codes were given an index value where 0 equals a low FI risk and 100 equals a high FI risk. Then, the values were ranked as 1 (low FI risk), 2 (mild FI risk), 3 and 4 (moderate FI risk), or 5 (high FI risk) [31, 32]. The FII across the zip codes included in this study are as follows: 89,101 (97.5; 5), 89,106 (98.0; 5) and 89,030 (97.3; 5), 89,031 (54.5; 3), and 89,032 (75.4; 4). For data analysis, we considered zip codes as *moderate FI risk* (89,031, 89,032) and *high FI risk* (89,101, 89,106, 89,030). The Open Source Geographic Information System (QGIS 3.26) generated the visual maps of nurturing care assets and the FII.

### Data analysis

Descriptive analyses were conducted using Microsoft Excel. First, we described the characteristics of community assets within each NCF component following the operational definitions provided in Table 1. Next, an exploratory analysis of the differences in the number of nurturing care assets across zip codes with moderate and high FI risk was conducted to characterize whether disparities across each NCF component are a function of FI risks.

## Results

### Characteristics of nurturing care assets

A total of 353 nurturing care assets were included in the CAM (Fig. 1). Among the NCF components, adequate nutrition had the most assets (*n* = 218, 61.8%), while responsive caregiving had the least (*n* = 26, 7.4%) (Table 2).

**Table 2:** Frequency and characteristics of nurturing care assets by food security needs across five under-resourced zip codes

Food Insecurity Index (FII)*	High Food Insecurity			Moderate Food Insecurity		Traveling Services (*n*)	Frequency (*n*)	Percentage (%)
Zip Codes	89,101	89,106	89,030	89,031	89,032			
**Total Nurturing Care Assets**	76	70	75	40	62	31	**353**	**100**
**Good Health**	6	16	12	3	8	3	**48**	**13.6**
Hospital/Urgent Care	-	4	2	2	2	1	11	22.9
Medical Clinic	2	4	3	-	4	-	13	27.1
Mental Health Center	-	-	1	-	-	1	2	4.2
Nonprofit Health Organizations	-	-	-	-	1	1	2	4.1
Outreach Clinic	-	-	1	-	-	-	1	2.1
Pediatric Health Clinic	2	3	2	-	-	-	7	14.6
Women’s Health Care	2	5	3	1	1	-	12	25.0
**Adequate Nutrition**	63	37	52	23	43	-	**218**	**61.8**
Community Garden	10	4	5	1	-	-	20	9.2
Convenience Store	23	12	27	10	24	-	96	44.0
Farmer’s Market	3	-	1	-	-	-	4	1.8
Food Distribution**	-	-	-	-	14	-	14	6.4
Food Pantry	9	12	8	4	-	-	33	15.1
Grocery Store	12	3	6	7	5	-	33	15.1
Food Assistance Program (WIC)	6	6	5	1	-	-	18	8.3
**Security & Safety**	5	8	8	3	6	-	**30**	**8.5**
Church	3	4	6	3	4	-	20	66.7
Community Center	-	-	1	-	-	-	1	3.3
Library	1	1	1	-	1	-	4	13.3
Police Station	-	1	-	-	-	-	1	3.3
Public Park	1	2	-	-	1	-	4	13.3
**Opportunities for Early Learning**	2	9	3	10	5	2	**31**	**8.8**
Childcare Financial Support	-	1	-	-	-	-	1	3.22
Daycare & Preschool	2	7	2	10	4	-	25	80.7
Nonprofit Education Program	-	1	1	-	1	2	5	16.1
**Responsive Caregiving**	-	-	-	-	-	26	**26**	**7.4**
Doula	-	-	-	-	-	13	13	50.0
Doula/Lactation Specialist	-	-	-	-	-	7	7	26.9
Home Visiting Program	-	-	-	-	-	2	2	7.7
Lactation Specialist	-	-	-	-	-	4	4	15.4

*Intensity of food insecurity needs was measured by the Food Insecurity Index by the Conduent Healthy Communities Institute available from: https://help.healthycities.org/hc/en-us/articles/1500001872902-What-is-the-Food-Insecurity-Index-ranking-and-how-is-it-determined-

**Food distribution refers to services that distribute food items by processes different than a food pantry (i.e. mobile food distribution and pop-up distribution)

#### Good health

The good health component consisted of 48 (13.6%) assets total, with the most prevalent type being medical clinics (*n* = 13, 27.1%), women’s health care centers (*n* = 12, 25.0%), and hospital and urgent cares (*n* = 11, 22.9%) (Table 2).

#### Adequate nutrition

The adequate nutrition component had the most assets of all the NCF components (*n* = 218, 61.8%). Convenience stores (*n* = 96, 44.0%), grocery stores (*n* = 33, 15.1%), and food pantries (*n* = 33, 15.1%) provided the most adequate nutrition assets, while the least provided asset were farmer’s markets (*n* = 4, 1.8%) (Table 2).

#### Security and safety

The security and safety component had a total of 30 (8.5%) assets. Churches (*n* = 20, 66.7%) were the most prevalent asset. Churches were available in every zip code, while community centers (*n* = 1) and police stations (*n* = 1) were in one zip code each (89,106 and 89,030, respectively) (Table 2).

#### Opportunities for early learning

The opportunities for early learning consisted of 31 (8.8%) assets. Among these assets, 80.7% comprised daycares and preschools (*n* = 25).

#### Responsive caregiving

Responsive caregiving assets (*n* = 26) are available to the zip code areas only via traveling services, with most being provided by doulas (*n* = 13) (Table 2).

### Nurturing care assets across food insecurity risk

**Fig. 2:**
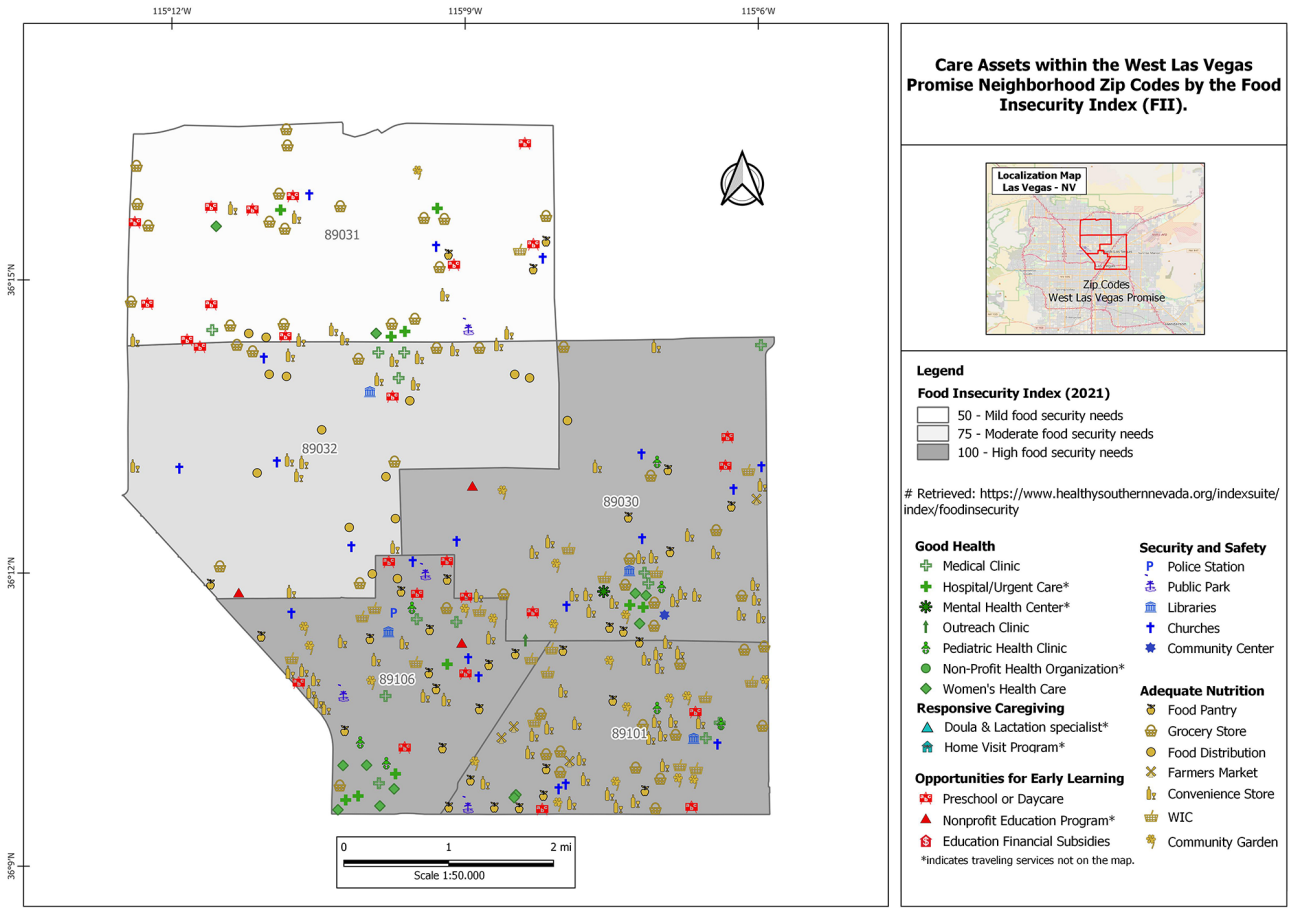
Community asset mapping

Zip codes with high FI risk had the most assets overall (*n* = 76, 75, and 71) compared to zip codes with moderate FI risk (*n* = 40 and 62) (Table 2). In the zip codes with high FI risk, there was a higher number of adequate nutrition and good health assets compared to zip codes with moderate FI risk. The adequate nutrition resources within these zip codes are densely clustered and mainly consist of convenience stores and food pantries. All food distribution sites were located within zip codes with moderate FI (Table 2). Zip codes with high FI risk share the most security and safety assets (*n* = 21 out of 30). We identified a low number of opportunities for early learning (two daycare and preschool assets) within zip codes with high FI risk compared to 10 located within zip codes with moderate FI. Zip codes with high FI risk did not have any responsive caregiving assets (Fig. 2).

## Discussion

To our knowledge, this study is one of the first to use CAM to characterize nurturing care assets across under-resourced zip codes with moderate and high FI risk. The distribution of nurturing care assets found in our study have implications for food security within the first 1,000 days of a child’s life in under-resourced zip codes. As expected, the CAM identified a large number of assets in zip codes with high FI risk, which correspond to the zip codes with lower income per capita. We observed that as FI risk decreased so did the number of nurturing care assets. Although there is no specific number of nurturing care assets that are recommended to improve populations health and well-being in areas with high FI risk, our analysis indicate the importance of characterizing existing assets and the type of service provided to identify barriers and opportunities to mitigate FI in a specific zip code. Thus, our CAM is one of the first studies providing insight into how zip codes with moderate and high FI risk can use the NCF as a theory to benefit a multi-sectoral approach to address FI and promote equity from pregnancy through early childhood [7, 8].

A large number of assets partaking in the adequate nutrition of the NCF was not expected across zip codes with high FI risk. Most of the adequate nutrition assets in our study were convenience stores, which have been shown the most common source of food purchase in low-income zip codes [33, 34]. Our finding is concerning because convenience stores have been known for not providing healthy or culturally appropriate food options [35–37]. Food pantries were also common in zip codes with high FI risk; while they are considered feasible and accessible evidence-based short-term solutions to relieve FI [35], the food distributed in pantries across the U.S has been described as low in nutritional value and high in saturated fats and often not culturally appropriate [36, 37]. The only exceptions were WIC clinics and community gardens & farmers’ markets. Available across all zip codes, WIC clinics stood out as the main source of maternal-child nutrition education. Prior evidence has demonstrated that WIC reduces the prevalence of FI among households with children by at least 20% and the prevalence of very low food security by at least 38%; furthermore, the risk of FI among children decreased with each additional WIC visit [38]. Community gardens and farmer’s markets were identified across zip codes with moderate and high FI risk and identified as healthy food assets. Community gardens and farmer’s markets have been described to increase access to fresh fruits and vegetables, reduce FI, and alleviate nutritional gaps, particularly in urban areas [39–41].

Disparities in good health, early learning, and security and safety assets were also observed in the context of high FI risk. For example, only one mental health center was found across all zip codes, and one was available via traveling services, but neither specialized in perinatal mental health. Maternal mental health support in zip code with moderate and high FI risk is critical because depression and anxiety are more frequent among food-insecure households, with ripple negative effects on parenting and responsive care [11]. Daycares or preschools were found in higher numbers in zip codes with moderate FI risk compared with high FI risk zip codes. The provision of high-quality and affordable childcare is critical to advancing gender equality and the advancement of women in the workforce [42]. In this context, a safe place to leave very young children while caregivers go to work or advance studies may contribute to an increase in household income which is critical to mitigating FI [43]. Churches and libraries were assets providing security and safety services to reduce FI. Faith-based community interventions [44] and library-based community interventions [45] have demonstrated success in addressing the social needs by providing food pantries and free-meals programs in under-resourced zip codes.

Our CAM identified a low number of responsive caregiving assets which corroborates to prior national and local levels analyses [46]. Because most of these assets were not located inside the zip codes, it was challenging to measure the available responsive caregiving assets across the zip codes [47]. To resolve this issue, we conducted a survey among responsive caregiving providers to gain insight as to where these individuals provide their care, and if they have worked in our research location. We acknowledge the survey may not capture the entire body of assets available to these zip codes, however this attempt has captured valuable information for this study. Other limitations of our CAM approach to inform asset-based interventions should be considered. First, our CAM does not include data on the relational nature between collected assets [48]. Because of this, our team is exploring opportunities to measure this relational dynamic among nurturing care assets participating in the co-creation of the nurturing care asset-based strategy (e.g., social network analysis) [49–51]. Second, interventions utilizing CAM pose a risk for two premises essentialization (i.e., failing to consider variations in identities, contexts, and cultures across zip codes) and reflexivity (i.e., assuming that zip codes are congruent with their geographical locations and boundaries) [52]. Therefore, we acknowledge these two premises may be present to some extent in the way data have been interpreted by our team. Third, the location-specific nature of the CAM can limit the applicability of the content to a larger audience. However, to minimize this, generalizing the steps we made throughout the process provides a basic framework for others to replicate it in other zip codes.

## Conclusion

Our CAM exercise followed a systematic search, validation, and categorization process, which provides strength to the evidence of the current nurturing care assets across five Las Vegas zip codes and can be replicable in projects trying to identify zip code-specific assets. A nurturing care asset-based strategy to address high FI risk requires (i) expansion of healthy food assets; and (ii) utilization of existing security and safety community spaces to deliver services missing across responsive care, early learning and perinatal mental health. By strengthening systems across existing nurturing care assets qualifying levels of care such as universal support (i.e., standard care available to all families), focused support (i.e., care for families with increased FI risk), and indicated support (i.e., care for families struggling with FI) [47] can be a comprehensive approach likely to decrease structural barriers and promote equity in food security.

### Electronic supplementary material

Below is the link to the electronic supplementary material.

Supplementary Material 1

Supplementary Material 2

Supplementary Material 3

## Data Availability

All data generated or analyzed during this study are included in this published article and its supplementary information files. The datasets used and analyzed during the current study are available from the corresponding author upon reasonable request.

## Abbreviations

FI: Food Insecurity
FII: Food Insecurity Index
NCF: Nurturing Care Framework
CAM: Community Asset Mapping
SNAP: Supplemental Nutrition Assistance Program
WIC: Supplemental Nutrition Program for Women, Infants, and Children
