# Supplementary material for: Nurturing care assets for food security: a community asset mapping approach
Source: J Health Popul Nutr. 2024 Jun 6;43:78. doi: 10.1186/s41043-024-00573-9 (PMC11157999; doi:10.1186/s41043-024-00573-9)
Supplement: Supplementary file 1 — Supplementary Material 1 [file 41043_2024_573_MOESM1_ESM.docx]

Appendix 2: Responsive caregiving survey questions.

*Introduction*: “You are invited to participate in a short survey discussing your activity as a practicing birth worker (i.e., doula, lactation consultant/counselor, midwife, etc.). The purpose of this survey is to understand the current prenatal, birth, and postpartum services provided in parts of North and West Las Vegas. This survey is part of a larger study being conducted to help pregnant people and toddlers in select zip codes.

This research has been approved by the UNLV Institutional Review Board (IRB) study number 1801320-EXE and is funded by the National Institute of Health (NIH) award number U01OD033239.

We thank you for your participation!”

*Questions:*

Q1) What is your name?

Q2) What is your race or ethnicity?

- American Indian or Alaskan Native
- Asian
- Black or African American
- Native Hawaiian or Pacific Islander
- Bi-racial / Mixed Ethnicity
- Hispanic or Latina/o/e/x
- Other-please specify

Q3) What is your gender?

- Male
- Female
- Non-Binary / Third Gender
- Transgender
- A gender identity not listed- please specify
- Prefer not to share

Q4) How can we contact you?

- Phone number
- Email
- Website
- Other

Q5) What services do you provide? (i.e. prenatal, lactation, postpartum, childbirth educator, etc.)

Q6) Are you practicing part time or full time?

- Part time
- Full time
- Not practicing
- Other-please specify

Q7) Do you speak a language(s) other than English? If yes, please provide the language.

- Yes- please specify
- No

Q8) What is your target population for services? (i.e. LGBTQIA+, BIPOC, etc.)

Q9) Do you take Health Insurance(s)? If yes, please provide which insurance.

- Yes- please specify
- No

Q10) Do you provide a sliding scale rate?

- Yes
- No
- Maybe

Q11) Do you provide scholarships for anyone who may not be able to pay for services?

Q12) Do you provide payment plans?

- Yes
- No
- Maybe

Q13) In the last six months/year how many clients have you had from the following zip codes:

- 89101
- 89106
- 89030
- 89031
- 89032
