# Supplementary material for: Nurturing care assets for food security: a community asset mapping approach
Source: J Health Popul Nutr. 2024 Jun 6;43:78. doi: 10.1186/s41043-024-00573-9 (PMC11157999; doi:10.1186/s41043-024-00573-9)
Supplement: Supplementary file 3 — Supplementary Material 3 [file 41043_2024_573_MOESM3_ESM.docx]

Appendix 1: Descriptive data of the five zip codes within the “West Las Vegas Promise Neighborhood”.

|  | **West Las Vegas Promise Zip Codes** | | | | |
| --- | --- | --- | --- | --- | --- |
|  | **89101** | **89106** | **89030** | **89031** | **89032** |
| **Total Population (*n*)** | 47,374 | 28,457 | 57,518 | 76,595 | 50,100 |
| **White, non-Hispanic** | 19,952 | 7,865 | 21,728 | 35,125 | 17,862 |
| **Black/African American, non-Hispanic** | 7,325 | 10,482 | 8,780 | 17,015 | 14,590 |
| **2+ Races, non-Hispanic** | 2,542 | 1,706 | 2,542 | 6,009 | 3,325 |
| **Some Other Races, non-Hispanic** | 15,638 | 6,844 | 22,712 | 11,648 | 10,376 |
| **Hispanic/Latino^*^** | 29,179 | 11,944 | 41,925 | 25,844 | 20,505 |
| **Median Household Income** | $29,363 | $33,859 | $45,049 | $76,579 | $67,286 |
| **Families with children living below poverty ^a^** | 1,699 (21.4%) | 1,091 (18.6%) | 2,254 (19.7%) | 989 (5.4%) | 777 (6.7%) |

Data collected from the Healthy Southern Nevada Demographic Dashboard: https://www.healthysouthernnevada.org/

*Not exclusive to other races/ethnicities

[^a^ Based on the Census Bureau household poverty threshold](about:blank)
